# Aggregation‐Induced Emission Luminogens Sensitized Quasi‐2D Hybrid Perovskites with Unique Photoluminescence and High Stability for Fabricating White Light‐Emitting Diodes

**DOI:** 10.1002/advs.202100811

**Published:** 2021-05-29

**Authors:** Yuanwei Wang, Dingyuan Yan, Lei Wang, Dong Wang, Ben Zhong Tang

**Affiliations:** ^1^ Center for AIE Research Shenzhen Key Laboratory of Polymer Science and Technology Guangdong Research Center for Interfacial Engineering of Functional Materials College of Materials Science and Engineering Shenzhen University Shenzhen 518060 P. R. China; ^2^ College of Physics and Optoelectronic Engineering Shenzhen University Shenzhen 518060 P. R. China; ^3^ Department of Chemistry, Hong Kong Branch of Chinese National Engineering Research Center for Tissue Restoration and Reconstruction, Institute of Molecular Functional Materials The Hong Kong University of Science and Technology, Clear Water Bay, Kowloon Hong Kong 999077 P. R. China

**Keywords:** aggregation‐induced emission, fluorescence resonance energy transfer, quasi‐2D hybrid perovskite, white LEDs

## Abstract

In order to endow quasi‐2D organic‐inorganic hybrid metal halide perovskites (quasi‐2D‐PVK) with superior performance, an aromatic organic ligand with aggregation‐induced emission (AIE) features is rationally designed and utilized for constructing distinctive quasi‐2D‐PVK materials. This AIE‐active ligand, TTPy‐NH_2_, well fits into the lattices of quasi‐2D‐PVK and leaves hydrophobic tails surrounding PVK layers, making the presented TTPy‐NH_2_/PVK film extraordinary in terms of both luminescence and stability. Benefiting from the prominent sensitization function and AIE tendency of TTPy‐NH_2_, the presented TTPy‐NH_2_/PVK film exhibits a high quantum yield of 62.2%, unique blue‐red dual‐emission property of both blue and red, high stability with the remnant of more than 94% fluorescence intensity remnant after 21 days. As a result, TTPy‐NH_2_/PVK film is capable of constituting high‐performance white light‐emitting diodes, with its color gamut reaching 138% of the National Television System Committee （NTSC） standard and the maximum efficiency is 105 lm W^−1^ at 20 mA. Evidently, a win‐win effect is achieved by the integration of AIE‐active ligands and quasi‐2D‐PVK, which are two of the most reputable solid‐state luminogens. This developed protocol thus opens up a new avenue for exploring the next generation of luminescent devices.

## Graphical abstract

An aggregation‐induced emission (AIE)‐featured organic ligand TTPy‐NH_2_ is rationally designed and successfully utilized for sensitizing quasi‐2D‐perovskites (PVK). The fabricated TTPY‐NH_2_/PVK film exhibits as high as 62.2% of quantum yield, unique dual‐emissions, and high stability, making it potential in fabricating high‐performance perovskite‐based white light‐emitting diodes.

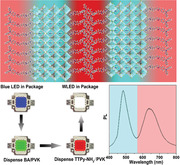

## 1 Introduction

Organic‐inorganic hybrid metal halide perovskites (PVK) have emerged as a burgeoning research field in the past few years.^[^ ^1^, ^2^, ^3^, ^4^ ^]^ Thanks to their inherent advantages of high photoluminescence (PL) quantum yield (up to 100%) in solution, tunable emission from visible to near‐infrared ranges, and high power conversion efficiency, PVK have been widely applied in solar cells, light‐emitting diodes (LEDs), photodetectors, sensing and so on.^[^ ^1^, ^2^, ^3^, ^4^, ^5^, ^6^, ^7^, ^8^, ^9^ ^]^ As a family member of the emerging PVK materials, quasi‐2D PVK (quasi‐2D‐PVK) has aroused great attention due to the excellent optical performance and appealing environmental stability, ascribing to their multi‐quantum‐wells structures.^[^ ^10^, ^11^, ^12^ ^]^ Quasi‐2D‐PVK layered materials have a general formula of (RNH_3_)_2_(CH_3_NH_3_)_n−1_A_n_X_3n+1_, in which R, A, and X represent aliphatic/aromatic ligands, metal cation (Pb^2+^, Sn^2+^) and halide (Cl^−^, Br^−^, I^−^), respectively. The variable “n” indicates the number of metal cation layers between two organic ligands layers.^[^ ^13^, ^14^, ^15^ ^]^ The orderly combined 2D orientation architecture of PVK and organic ligands constituents provides more structural and qualitative versatility through introducing different organic ligands into the quasi‐2D‐PVK frameworks, which play a crucial role in sensitizing quasi‐2D‐PVK. Evidently, the exploration of appropriate organic ligands is supremely significant for fabricating high‐performance quasi‐2D‐PVK. Although many types of organic ligands, of particular aliphatic ammonium salt cations, have been utilized in quasi‐2D‐PVK sensitization, the current situation is still far from ideal. Furthermore, quasi‐2D‐PVK materials are generally constructed through solution method, but as practically utilized in the solid‐state, where the property is apparently different from that of originally prepared counterpart. The solidification process could cause various issues including crystal structure distortion, ligands detachment, and interface interaction, which would seriously hamper their optical performance. Therefore, developing new kinds of multifunctional ligands sharing excellent sensitization property and inherent luminescent behavior, which could fabricate quasi‐2D‐PVK materials with superior properties, especially in solid‐state, remains unexploited and supremely desirable.

Given the circumstances, luminogens with aggregation‐induced emission (AIE) tendency could be extraordinary candidates. AIE refers to a unique phenomenon that some luminogens are barely emissive in the molecularly dissolved state, but they emit intensively in aggregates resulting from the highly suppressed non‐radiative decay due to restriction of intramolecular motions in the aggregation state. The AIE features endow these luminogens with intrinsic characteristics, including high emission brightness in solid, high photobleaching threshold, and great tolerance for any concentrations.^[^ ^16^, ^17^, ^18^ ^]^

**Figure 1:**
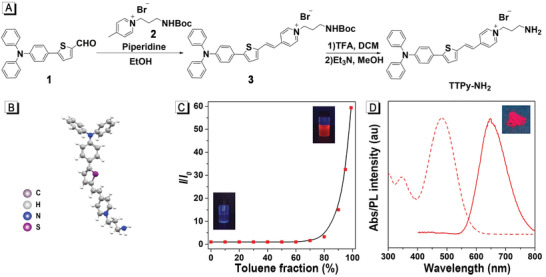
Synthesis and characteristics of the TTPy‐NH_2_ molecule. A) Synthetic route of TTPy‐NH_2_. B) Molecular model of TTPy‐NH_2_ (carbon, gray; hydrogen, silver; nitrogen, blue; sulfur, purple). C) Plots of the relative emission intensity (*I*/*I*_0_) versus toluene fraction. *I*_0_ and *I* are the peak values of fluorescence intensities of TTPy‐NH_2_ in DMSO and DMSO/toluene mixtures with different *f*_T_. D) Absorption spectra of TTPy‐NH_2_ in DMSO and emission spectra of TTPy‐NH_2_ powder. Inset shows pictures of TTPy‐NH_2_ powder under 365 nm excitation.

In this work, we elaborately designed an AIE‐active organic ligand, namely TTPy‐NH_2_ (**Figure** **1**), working as an interlayer for constructing efficient quasi‐2D‐PVK materials, which exhibit unique luminescent properties and high environmental resistance, especially in form of film. Interestingly, benefiting from the dual‐emission characteristics originated from both TTPy‐NH_2_ and PVK, the presented TTPy‐NH_2_/PVK film exhibits great potential in fabricating new generation of perovskite‐based white light‐emitting diodes (Pe‐wLEDs).

## 2 Results and Discussion

TTPy‐NH_2_ consists of an amino head, rod‐like architecture, and hydrophobic tail, shaped as a dart. We hypothesize that protonated amino head could serve as an anchor to coordinate with PVK layers through the hydrogen bond between protonated amino‐terminal and PbBr_6_^−^ octahedron. The rod‐shaped skeleton is beneficial to fit into quasi‐2D‐PVK lattices well. Moreover, the hydrophobic triphenylamine tail of TTPy‐NH_2_ could form a compact water‐proof outer layer for PVK layers. Concurrently, the intramolecular motions of TTPy‐NH_2_ would be greatly restricted when being intensely compacted in the space between PVK layers, consequently providing amplified fluorescence intensity. To verify these hypotheses, we started with the synthesis and characterization of TTPy‐NH_2_. A simple synthetic strategy involving a few steps was proposed (Figure 1A). Briefly, the amino group protected intermediate, TTPy‐NHBoc, was readily obtained by a one‐step condensation reaction with a yield of 82.4%. Trifluoroacetic acid and triethylamine were then respectively used to remove the protecting group and release the naked amino part. The AIE properties of TTPy‐NH_2_ were further investigated by PL spectroscopies in DMSO/toluene solvent system with different fractions of toluene, using DMSO as the good solvent, and toluene as the poor solvent. As illustrated in Figure 1C and Figure S10, Supporting Information, the PL intensity eventually increased to 60‐fold upon the formation of aggregates when the toluene faction (*f*_T_) increased to 99% comparing with that in pure DMSO, solidly indicating the typical AIE behavior, which was also verified by the correspondingly raised quantum yield from 0.2 to 10.6%. In addition, the absorption peak of TTPy‐NH_2_ in DSMO and emission peak of TTPy‐NH_2_ powder were centered at 482 and 656 nm, respectively (Figure 1D, Table S1, Supporting Information).

**Figure 2:**
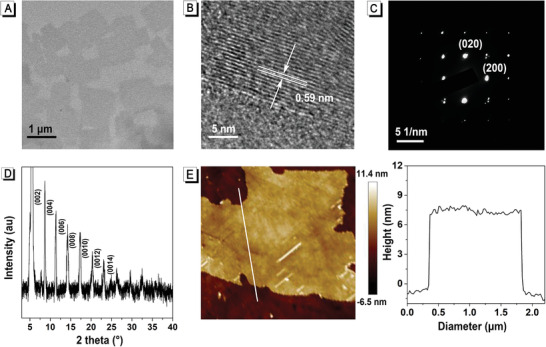
Fabrication and characteristics of the TTPy‐NH_2_/PVK sheet. A) TEM image, B) HRTEM, C) SAED image, D) XRD pattern, and E) AFM image and height profile of TTPy‐NH_2_/PVK sheets.

By modulating the proportion of raw materials, TTPy‐NH_2_/PVK sheets with different values of n (n = 1, 2, 3, 4) were prepared via the modified ligand‐assisted reprecipitation (LARP) method. LARP is a well‐established synthetic route to produce PVK, which relies on the solubility difference of the PVK in a polar precursor solution and a weakly polar precipitant solvent.^[^ ^19^ ^]^ The LARP involves the nucleation trigger and growth of PVK nanocrystals, where the confinement effect of ligand plays an important role in the supersaturation process of the PVK solution. To determine the crystal structure of TTPy‐NH_2_/PVK, transmission electron microscopy (TEM), high‐resolution transmission electron microscopy (HRTEM), X‐ray diffraction (XRD), and atomic force microscope (AFM) were utilized as demonstrated in **Figure** **2**. As shown in Figure 2A, TTPy‐NH_2_/PVK grew on a lacy carbon grid appeared as ultrathin square sheets with an average edge length of ≈0.6 µm, ranging from 0.1 to 2 µm. Carbon, lead, and sulfur (from TTPy‐NH_2_) were all found to be present in the square as shown in the elemental distribution patterns (Figure S11, Supporting Information), which confirmed the existence of TTPy‐NH_2_ and PVK in the composite sheet. The HRTEM image shown in Figure 2B displayed the lattice fringes of TTPy‐NH_2_/PVK with an average *d*‐spacing of ≈0.59 nm, which was consistent with (001) planes of cubic phases of the 3D perovskites CH_3_NH_3_PbBr_3_. The in‐plane structural information was further revealed by selected‐area electron diffraction (SAED), as depicted in Figure 2C. The calculated average in‐plane lattice constants *a* and *b* were 5.82 and 5.88 Å, respectively, which were similar to the lattice constants in the bulk crystals CH_3_NH_3_PbBr_3_,^[^ ^20^ ^]^ further confirming the crystal structure of the TTPy‐NH_2_/PVK sheets. The XRD pattern of the thin film sample was recorded and shown in Figure 2D. The (00*l*) diffraction peaks can be obviously observed in the XRD pattern, which suggests the existence of the layered crystalline structures of 2D TTPy‐NH_2_/PVK sheets. Additionally, the out‐of‐plane layer spacing was calculated from the XRD pattern to be 4.1 nm by using Braggs’ law, which was consistent with layer spacing of ≈4.3 nm observed from the TEM image in Figure S12, Supporting Information. Besides, the thickness of these square sheets was quantified to be ≈7.2 nm (±0.2 nm) in average, as displayed in the AFM pattern in Figure 2E.

**Figure 3:**
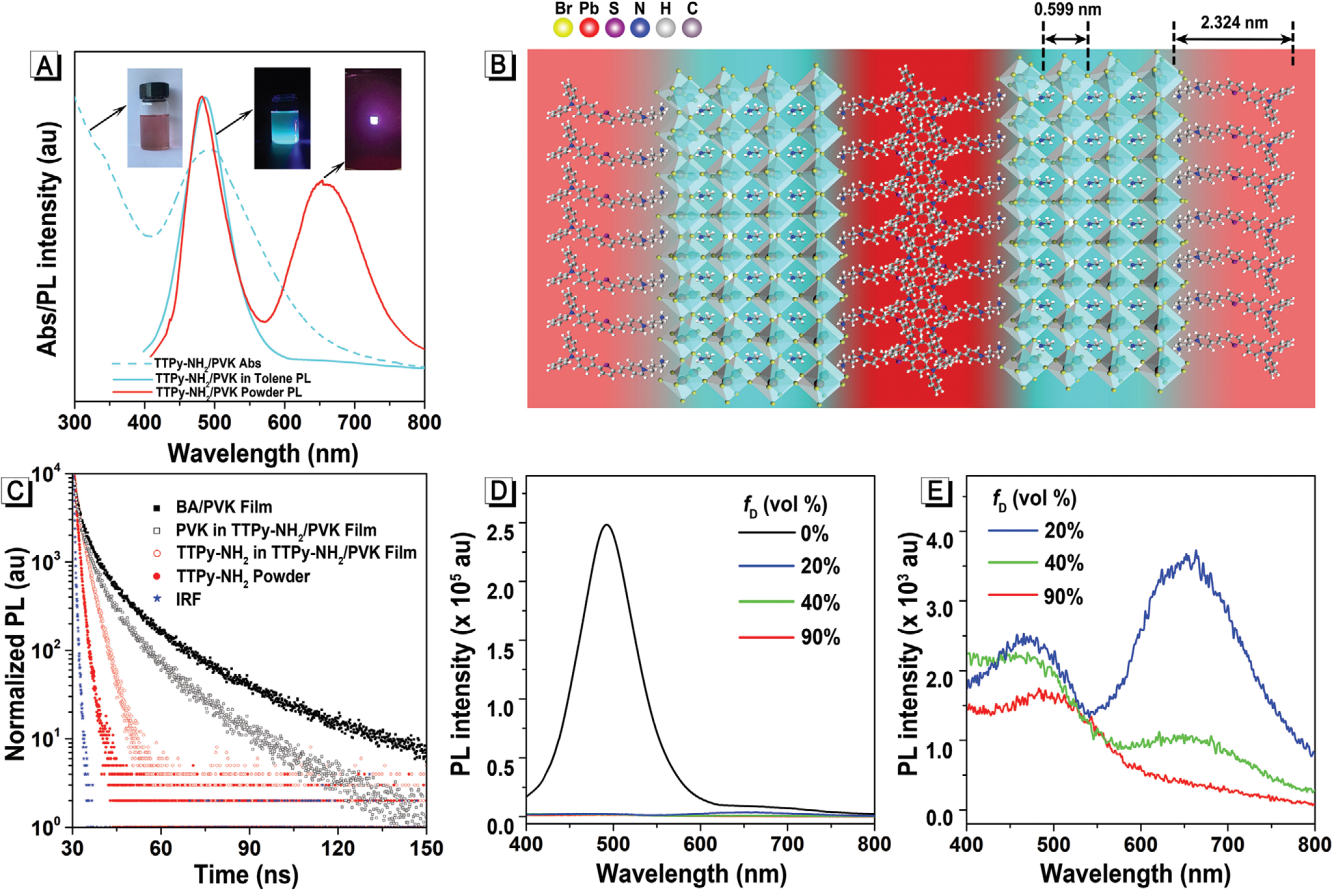
Optical and structural characteristics of TTPy‐NH_2_/PVK sheet. A) Absorption and emission spectra of TTPy‐NH_2_/PVK (n = 4) nanosuspension and the corresponding film. Inset shows pictures under 365 nm excitation. B) Schematic model of TTPy‐NH_2_/PVK sheet (n = 4). C) TRPL spectra of BA/PVK film (black solid), PVK in TTPy‐NH_2_/PVK film (black hollow), TTPy‐NH_2_ powder (red solid), and TTPy‐NH_2_ in TTPy‐NH_2_/PVK film (red hollow). D) PL spectra of TTPy‐NH_2_/PVK nanosuspension in DMSO/toluene with different DMSO fractions (*f*_D_); Concentration: 10 µm; *λ*ex: 365 nm. E) Enlarged spectra of TTPy‐NH_2_/PVK nanosuspension in DMSO/toluene when *f*_D_ = 20, 40, and 90%.

By modulating the proportion of raw materials, TTPy‐NH_2_/PVK sheets with different values of n (n = 1, 2, 3, 4) were prepared respectively. Meanwhile, butylammonium (BA) sensitized PVK sheets (BA/PVK) were prepared in a similar way as control. BA is a kind of short‐chain alkane serving as a non‐fluorescent ligand molecule that has been proved to be effective for PVK fabrication.^[^ ^21^, ^22^ ^]^ An absorption peak at 382 nm was observed in the TTPy‐NH_2_/PVK (n = 1) suspension and the corresponding band gap was calculated to be 3.24 eV. The PL emission maximum of TTPy‐NH_2_/PVK (n = 1) was located at 436 nm under 365 nm irradiation, exhibiting bright blue emission (Figure S13A, Supporting Information). As depicted from the optical spectra of TTPy‐NH_2_/PVK suspension with different PVK layers number n (Figure S13B,C, Supporting Information), the emission maximum of TTPy‐NH_2_/PVK (n = 4) gradually red‐shifted to 473 nm along with the increase of n (**Figure** **3A**), which was a typical quantum effect of quasi‐2D‐PVK.^[^ ^23^, ^24^, ^25^ ^]^ TTPy‐NH_2_/PVK (n = 4) nanosuspension emitted blue fluorescence and possessed a high quantum yield of 56.4%. As for the absorption spectrum, apart from the absorption band below ≈400 nm corresponding to the bandgap absorption of PVK, there was another absorption band at 400–550 nm in all the samples of TTPy‐NH_2_/PVK (n = 1, 2, 3, 4), while it was absent in the sample of BA/PVK (Figure S13B, Supporting Information), revealing the existence of TTPy‐NH_2_ in the samples of TTPy‐NH_2_/PVK.

Given the fact that the emission peak of PVK overlapped with the absorption band of TTPy‐NH_2_ to a great extent (Figure S14, Supporting Information), meanwhile, PVK and TTPy‐NH_2_ were confined within 10 nm, which all became prerequisites for effective fluorescence resonance energy transfer (FRET) process between PVK (as donor) and TTPy‐NH_2_ (as accepter).^[^ ^26^ ^]^ However, the emission peak of TTPy‐NH_2_ centered at ≈656 nm cannot be directly observed in the PL spectrum of TTPy‐NH_2_/PVK nanosuspension, while it was obvious in the spectra of TTPy‐NH_2_/PVK films (Figure 3A). Presuming the AIE features of TTPy‐NH_2_, its aggregation state surrounding PVK was supposed to be the key point for the emission distinction. The schematic model of TTPy‐NH_2_/PVK was proposed and demonstrated in Figure 3B. While in toluene solution, TTPy‐NH_2_/PVK sheets were more likely to be monodisperse, resulting in loose packing of TTPy‐NH_2_ in the outer layer of PVK with amino head anchored into PVK lattices, which induced weak emission of TTPy‐NH_2_ (pale red emission in the schematic model) and that was too weak to display in the spectrum. In this condition, the energy transferred from PVK to TTPy‐NH_2_ mostly vanished in manners of kinetics and thermodynamics. As for TTPy‐NH_2_/PVK films, the intense and compact packing of TTPy‐NH_2_ layers significantly induced luminescence enhancement (dark red emission in the schematic model), the energy transferred from PVK to TTPy‐NH_2_ was efficiently irradiated as fluorescence. As a result, thanks to the highly amplified excited energy consumption of TTPy‐NH_2_ through radiative decay in the aggregated state, the quantum yield of TTPy‐NH_2_/PVK film was elevated to 62.2%, which was 5.8% higher than that of TTPy‐NH_2_/PVK nanosuspension. To further confirm the contribution of TTPy‐NH_2_ towards the high fluorescence efficiency, the corresponding proportion of PVK was mostly excluded by changing the excitation irradiation to 482 nm. As summarized in Table S2, Supporting Information, the quantum yield of TTPy‐NH_2_/PVK film was determined to be 29.5%, which was higher than that of TTPy‐NH_2_/PVK nanosuspension (24.3%), revealing that the irradiation contribution of TTPy‐NH_2_ was attributed to the aggregated state. The compact aggregation of TTPy‐NH_2_ layers was further proven through simulating layer‐spacing calculation. As shown in Figure 3B, TTPy‐NH_2_ molecule was calculated to be 2.324 nm in length when it was at the lowest energy state. As a result, the layer distance was supposed to be 4.648 nm (2 × 2.324 nm) if there was no overlap between the next two TTPy‐NH_2_ layers. However, according to the outcomes of the XRD pattern (Figure 2D) and TEM image (Figure S12, Supporting Information), the practical layer‐spacing was 4.3 nm, which was ≈13% shorter than the theoretical value, implying that some extent of overlapping, compression or inclination existed in the packing layers, which enhanced the aggregation degree of TTPy‐NH_2_ molecules.

Direct evidence for the dynamics of exciton transfer between PVK and TTPy‐NH_2_ was further provided by time‐resolved PL (TRPL) spectroscopy, as shown in Figure 3C. For simplicity, BA/PVK film sensitized by non‐fluorescent BA and TTPy‐NH_2_ powder were regarded as neat donor and neat acceptor, respectively. The lifetime of TTPy‐NH_2_, *τ*_accepter_, increased from 0.46 ns to 0.77 ns when surrounding 2D‐PVK layers and the lifetime of the donor PVK, *τ*_donor_, significantly decreased from 24.39 ns in neat donor to 20.57 ns (Table S3, Supporting Information), which strongly supported the FRET process between PVK and TTPy‐NH_2_. FRET is well‐known as a sensitive tool that can reflect distance change in nanoscale. The boosted emission of TTPy‐NH_2_ in TTPy‐NH_2_/PVK film was supposed to be the result of the AIE feature of TTPy‐NH_2_ and the enhanced FRET process due to the shortened distance between PVK and TTPy‐NH_2_.

Despite the superior luminescent properties of TTPy‐NH_2_/PVK endowed with the AIE‐active ligand TTPy‐NH_2_, it is crucial to further investigate their stability since that PVK would get easily damaged in the presence of moisture, thermal, and high energy radiation conditions under operating conditions, which seriously hindered their practical application.^[^ ^27^, ^28^ ^]^ In the following context, their stability investigation was separated into solvent resistance and environmental resistance, especially temperature and humidity resistance. DMSO is a well‐known good solvent of PVK which would decompose PVK by permeating into their crystal structure. Inevitably, the emission of PVK sharply declined along with the addition of DMSO (Figure 3D), which was consistent with conventional ligands‐assisted quasi‐2D‐PVK materials, BA/PVK, where the decline of fluorescence was precipitous as well (Figure S15A, Supporting Information). Impressively, the emission PVK of TTPy‐NH_2_/PVK still maintained when the DMSO faction was 90% (Figure 3E), while BA/PVK has completely lost its fluorescence by employing only 20% of the DMSO faction (Figure S15B, Supporting Information), evidently suggesting the much stronger interaction of TTPy‐NH_2_ and 2D‐PVK than that of BA and 2D‐PVK, which brings up their relatively better resistance to strong polarity solvent. More intriguingly, when 20% volume fraction of DMSO was added into TTPy‐NH_2_/PVK nanosuspension in toluene, the emission peak of TTPy‐NH_2_ at ≈656 nm appeared (Figure 3E), and its emission intensity gradually decreased along with the increase of DMSO, which was in similar tendency of TTPy‐NH_2_ in the DMSO/toluene solvent system shown in Figure 1C.

**Figure 4:**
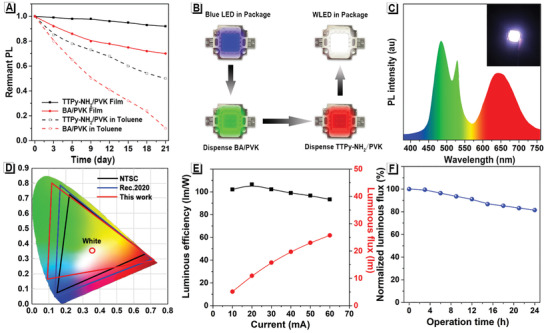
Fabrication and EL performance of the TTPy‐NH_2_/PVK‐based Pe‐wLED. A) The remnant PL intensity of TTPy‐NH_2_/PVK and BA/PVK nanosuspension/film after exposure in a humid environment for 21 days, measured every three days. B) Illustration of fabricating Pe‐wLEDs by utilizing TTPy‐NH_2_/PVK and BA/PVK as converters. C) EL spectrum, photograph (inset) and D) the corresponding CIE coordinate (red line) of Pe‐wLEDs device. E) Current‐dependent luminous efficiency and luminous flux of the Pe‐wLEDs device. F) Time‐dependent luminous flux of the Pe‐wLEDs device under continuous operation.

The environmental resistance ability of TTPy‐NH_2_/PVK was revealed by respectively placing TTPy‐NH_2_/PVK nanosuspension in toluene and films in an atmosphere both under the conditions of 30 ℃ of average temperature and 70% average humidity for 21 days, BA/PVK samples were also investigated in the same way as control. Their remnant PL was recorded and shown in **Figure** **4A**. As expected, TTPy‐NH_2_/PVK films performed much better with 94% of fluorescence intensity retained with negligible fluctuation of PL emission maximum (Figure S16, Supporting Information). The hydrophobic triphenylamine tails of TTPy‐NH_2_ molecules provided an ideal water‐proof outer layer for PVK layers, especially when TTPy‐NH_2_ packs around PVK layers in intensely aggregated states, which was not available for BA/PVK samples with short‐chain alkane in the outer layers.

The development of high‐performance wLEDs is of high importance in the fields of general illumination.^[^ ^29^, ^30^, ^31^ ^]^ Conventionally, combing chips with phosphors of three primary colors, such as BaMg_2_Al_16_O_27_:Eu^2+^ for blue, MgAl_11_O_19_:Ce^3+^,Tb^3+^ for green, and K_2_SiF_6_:Mn^4+^ for red, is the most popular method of preparing commercial wLEDs. To date, various PVK materials have been reported in fabricating wLEDs due to their high luminescent efficiency.^[^ ^32^, ^33^, ^34^ ^]^ In this work, high‐performance Pe‐wLEDs were fabricated by using TTPy‐NH_2_/PVK (n = 4) films as a double‐color converter due to their dual‐emissions of both blue and red, and BA/PVK was used as a green converter (Figure S13B, Supporting Information). As shown in Figure 4b. four 365 nm blue LED chips were packaged in a 10 mm × 10 mm LED unit in the first stage. Next, BA/PVK was mixed with silicone resin and dispensed into the package. After curing for 2 h at 100 °C in an oven, TTPy‐NH_2_/PVK film was attached on the top of the package as a blue and red color converter to achieve a Pe‐wLED, which featured a proven on‐chip PVK device design. As a result, the prepared Pe‐wLED emitted bright white luminance when it was lit up by external current injection (Figure 4C inset). The electroluminescence (EL) spectrum of the device demonstrated the presence of three primary peaks located at 473, 520, and 656 nm, where both blue and red fluorescence were supplied by TTPy‐NH_2_/PVK and green fluorescence was supplied by BA/PVK (Figure 4C). Attributed to the exceptional rendering of blue, green, and red color, the TTPy‐NH_2_/PVK‐based Pe‐wLEDs were endowed with a high color rendering index (CRI) of 89. Furthermore, the external quantum efficiency and the correlated color temperature were recognized as 4.8% and 6173 K, respectively. The corresponding Commission Internationale de l'Eclairage (CIE) coordinate was located at (0.329, 0.353), which ranged at a comparable stage with other white‐emitting PVK‐based LEDs, as shown in Table S4, Supporting Information. As shown in Figure 4D, the color gamut of the prepared Pe‐wLED illustrated a large color space of 138% of the National Television System Committee （NTSC） standard and 92% of Rec. 2020, which is the most important standard of the next generation 8K4K displays.^[^ ^35^ ^]^ Impressively, the color coordinates of the red (0.716, 0.284) points were very close to that defined by Rec. 2020 (0.708, 0.292), demonstrating the superiority of TTPy‐NH_2_/PVK based Pe‐wLEDs in displays.

Due to the instability of the PVK under high‐energy blue light illumination, most reported Pe‐wLEDs only exhibited a low luminous efficiency at around 50 lm W^−1^, which was far from the practical use in room lighting. The current‐dependent luminous efficiency and flux of the prepared Pe‐wLEDs are shown in Figure 4E. The maximum efficiency was 105 lm W^−1^ at 20 mA and the luminous efficiency was still higher than 95 lm W^−1^ when the current increased to 60 mA, indicating the excellent luminous performance at different drive currents. What's more, the prepared Pe‐wLED exhibited excellent luminescent stability, where more than 80% of the luminous flux was maintained even after continuous operation for 24 h (Figure 4F).

## 3 Conclusion

In summary, a typically AIE‐featured molecule TTPy‐NH_2_ has been synthesized and applied in sensitizing quasi‐2D‐PVK as multifunctional ligands. The obtained TTPy‐NH_2_/PVK exhibits up to 62.2% of quantum yield in solid film state and good PL stability in a humid and warm environment. Additionally, TTPy‐NH_2_/PVK films exhibit dual‐emission of both TTPy‐NH_2_ (red) and PVK (blue) due to the AIE feature of TTPy‐NH_2_ and the FRET process between PVK and TTPy‐NH_2_, which makes it available for high‐performance Pe‐wLEDs fabrication. The results presented here open up an avenue towards advanced luminescence materials for constructing high‐performance photoelectric devices.

## Conflict of Interest

The authors declare no conflict of interest.

## Supporting information

Supporting Information

## Data Availability

Data available on request from the authors.

## References

[1] Y. Hou , X. Du , S. Scheiner , D. P. McMeekin , Z. Wang , N. Li , M. S. Killian , H. Chen , M. Richter , I. Levchuk , N. Schrenker , E. Spiecker , T. Stubhan , N. A. Luechinger , A. Hirsch , P. Schmuki , H.‐P. Steinrück , R. H. Fink , M. Halik , H. J. Snaith , C. J. Brabec , Science 2017, 358, 1192. 29123021 10.1126/science.aao5561

[2] S. Yakunin , L. Protesescu , F. Krieg , M. I. Bodnarchuk , G. Nedelcu , M. Humer , G. D. Luca , M. Fiebig , W. Heiss , M. V. Kovalenko , Nat. Commun. 2015, 6, 8056. 26290056 10.1038/ncomms9056 PMC4560790

[3] G. Xing , B. Wu , X. Wu , M. Li , B. Du , Q. Wei , J. Guo , E. K. L. Yeow , T. C. Sum , W. Huang , Nat. Commun. 2017, 8, 14558. 28239146 10.1038/ncomms14558 PMC5333353

[4] A. Kojima , K. Teshima , Y. Shirai , T. Miyasaka , J. Am. Chem. Soc. 2009, 131, 6050. 19366264 10.1021/ja809598r

[5] X. Li , D. Bi , C. Yi , J.‐D. Décoppet , J. Luo , S. M. Zakeeruddin , A. Hagfeldt , M. Grätzel , Science 2016, 353, 58. 27284168 10.1126/science.aaf8060

[6] H. Tan , A. Jain , O. Voznyy , X. Lan , F. P. García de Arquer , J. Z. Fan , R. Quintero‐Bermudez , M. Yuan , B. Zhang , Y. Zhao , F. Fan , P. Li , L. N. Quan , Y. Zhao , Z.‐H. Lu , Z. Yang , S. Hoogland , E. H. Sargent , Science 2017, 355, 722. 28154242 10.1126/science.aai9081

[7] W. S. Yang , J. H. Noh , N. J. Jeon , Y. C. Kim , S. Ryu , J. Seo , S. I. Seok , Science 2015, 348, 1234. 25999372 10.1126/science.aaa9272

[8] W. S. Yang , B.‐W. Park , E. H. Jung , N. J. Jeon , Y. C. Kim , D. U. Lee , S. S. Shin , J. Seo , E. K. Kim , J. H. Noh , S. I. Seok , Science 2017, 356, 1376. 28663498 10.1126/science.aan2301

[9] H. Zhou , Q. Chen , G. Li , S. Luo , T.‐b. Song , H.‐S. Duan , Z. Hong , J. You , Y. Liu , Y. Yang , Science 2014, 345, 542. 25082698 10.1126/science.1254050

[10] I. C. Smith , E. T. Hoke , D. Solis‐Ibarra , M. D. McGehee , H. I. Karunadasa , Angew. Chem., Int. Ed. 2014, 53, 11232. 10.1002/anie.201406466 25196933

[11] D. H. Cao , C. C. Stoumpos , O. K. Farha , J. T. Hupp , M. G. Kanatzidis , J. Am. Chem. Soc. 2015, 137, 7843. 26020457 10.1021/jacs.5b03796

[12] H. Tsai , W. Nie , J.‐C. Blancon , C. C. Stoumpos , R. Asadpour , B. Harutyunyan , A. J. Neukirch , R. Verduzco , J. J. Crochet , S. Tretiak , L. Pedesseau , J. Even , M. A. Alam , G. Gupta , J. Lou , P. M. Ajayan , M. J. Bedzyk , M. G. Kanatzidis , A. D. Mohite , Nature 2016, 536, 312. 27383783 10.1038/nature18306

[13] D. B. Mitzi , Prog. Inorg. Chem. 1999, 48, 1.

[14] D. B. Mitzi , J. Chem. Soc., Dalton Trans. 2001, 1, 1.

[15] G. Lanty , K. Jemli , Y. Wei , J. Leymarie , J. Even , J.‐S. Lauret , E. Deleporte , J. Phys. Chem. Lett. 2014, 5, 3958. 26276477 10.1021/jz502086e

[16] Y. Wang , H. Li , D. Wang , B. Z. Tang , Adv. Funct. Mater. 2021, 31, 2006952.

[17] N. Song , Z. Zhang , P. Liu , Y.‐W. Yang , L. Wang , D. Wang , B. Z. Tang , Adv. Mater. 2020, 32, 2004208. 10.1002/adma.202004208 33150632

[18] J. Huang , B. He , Z. Zhang , Y. Li , M. Kang , Y. Wang , K. Li , D. Wang , B. Z. Tang , Adv. Mater. 2020, 32, 2003382. 10.1002/adma.202003382 32761671

[19] F. Zhang , H. Zhong , C. Chen , X.‐g. Wu , X. Hu , H. Huang , J. Han , B. Zou , Y. Dong , ACS Nano 2015, 9, 4533. 25824283 10.1021/acsnano.5b01154

[20] Y. Lei , Y. Chen , Y. Gu , C. Wang , Z. Huang , H. Qian , J. Nie , G. Hollett , W. Choi , Y. Yu , N. Kim , C. Wang , T. Zhang , H. Hu , Y. Zhang , X. Li , Y. Li , W. Shi , Z. Liu , M. J. Sailor , L. Dong , Y. Lo , J. Luo , S. Xu , Adv. Mater. 2018, 30, 1705992. 10.1002/adma.201705992 29611280

[21] X. Zhang , X. Ren , B. Liu , R. Munir , X. Zhu , D. Yang , J. Li , Y. Liu , D.‐M. Smilgies , R. Li , Z. Yang , T. Niu , X. Wang , A. Amassian , K. Zhao , S. Liu , Energy Environ. Sci. 2017, 10, 2095.

[22] C. M. M. Soe , C. C. Stoumpos , M. Kepenekian , B. Traoré , H. Tsai , W. Nie , B. Wang , C. Katan , R. Seshadri , A. D. Mohite , J. Even , T. J. Marks , M. G. Kanatzidis , J. Am. Chem. Soc. 2017, 139, 16297. 29095597 10.1021/jacs.7b09096

[23] S. Kumar , J. Jagielski , S. Yakunin , P. Rice , Y.‐C. Chiu , M. Wang , G. Nedelcu , Y. Kim , S. Lin , E. J. G. Santos , M. V. Kovalenko , C.‐J. Shih , ACS Nano 2016, 10, 9720. 27684448 10.1021/acsnano.6b05775

[24] C. C. Stoumpos , D. H. Cao , D. J. Clark , J. Young , J. M. Rondinelli , J. I. Jang , J. T. Hupp , M. G. Kanatzidis , Chem. Mater. 2016, 28, 2852.

[25] L. Dou , A. B. Wong , Y. Yu , M. Lai , N. Kornienko , S. W. Eaton , A. Fu , C. G. Bischak , J. Ma , T. Ding , N. S. Ginsberg , L.‐W. Wang , A. P. Alivisatos , P. Yang , Science 2015, 349, 1518. 26404831 10.1126/science.aac7660

[26] C.‐K. Lim , M. Maldonado , R. Zalesny , R. Valiev , H. Ågren , A. S. L. Gomes , J. Jiang , R. Pachter , P. N. Prasad , Adv. Funct. Mater. 2020, 30, 1909375.

[27] Y. Han , S. Meyer , Y. Dkhissi , K. Weber , J. M. Pringle , U. Bach , L. Spiccia , Y.‐B. Cheng , J. Mater. Chem. A 2015, 3, 8139.

[28] F. Li , M. Liu , J. Mater. Chem. A 2017, 5, 15447.

[29] K. Lin , J. Xing , L. N. Quan , F. P. G. de Arquer , X. Gong , J. Lu , L. Xie , W. Zhao , D. Zhang , C. Yan , W. Li , X. Liu , Y. Lu , J. Kirman , E. H. Sargent , Q. Xiong , Z. Wei , Nature 2018, 562, 245. 30305741 10.1038/s41586-018-0575-3

[30] H. Zhu , C. C. Lin , W. Luo , S. Shu , Z. Liu , Y. Liu , J. Kong , E. Ma , Y. Cao , R.‐S. Liu , X. Chen , Nat. Commun. 2014, 5, 4312. 25002064 10.1038/ncomms5312

[31] E. Song , Y. Zhou , X.‐B. Yang , Z. Liao , W. Zhao , T. Deng , L. Wang , Y. Ma , S. Ye , Q. Zhang , ACS Photonics 2017, 4, 2556.

[32] Y. Shirasaki , G. J. Supran , M. G. Bawendi , V. Bulović , Nat. Photonics 2013, 7, 13.

[33] K.‐J. Chen , H.‐C. Chen , K.‐A. Tsai , C.‐C. Lin , H.‐H. Tsai , S.‐H. Chien , B.‐S. Cheng , Y.‐J. Hsu , M.‐H. Shih , C.‐H. Tsai , H.‐H. Shih , H.‐C. Kuo , Adv. Funct. Mater. 2012, 22, 5138.

[34] G. Nedelcu , L. Protesescu , S. Yakunin , M. I. Bodnarchuk , M. J. Grotevent , M. V. Kovalenko , Nano Lett. 2015, 15, 5635. 26207728 10.1021/acs.nanolett.5b02404 PMC4538456

[35] H.‐C. Wang , S.‐Y. Lin , A.‐C. Tang , B. P. Singh , H.‐C. Tong , C.‐Y. Chen , Y.‐C. Lee , T.‐L. Tsai , R.‐S. Liu , Angew. Chem., Int. Ed. 2016, 55, 7924. 10.1002/anie.201603698 27239980

