# Supplementary material for: Aggregation‐Induced Emission Luminogens Sensitized Quasi‐2D Hybrid Perovskites with Unique Photoluminescence and High Stability for Fabricating White Light‐Emitting Diodes
Source: Adv Sci (Weinh). 2021 May 29;8(15):2100811. doi: 10.1002/advs.202100811 (PMC8336619; doi:10.1002/advs.202100811)
Supplement: Supplementary file 1 — Supporting Information [file ADVS-8-2100811-s001.pdf]

© 2021 Wiley-VCH GmbH

ADVANCED SCIENCE Open Access

Supporting Information

for *Adv. Sci.*, DOI: 10.1002/advs.202100811

Aggregation-Induced Emission Luminogens Sensitized Quasi-2D Hybrid Perovskites with Unique Photoluminescence and High Stability for Fabricating White Light-Emitting Diodes

Yuanwei Wang, Dingyuan Yan, Lei Wang, Dong Wang and Ben Zhong Tang**

© 2021 Wiley-VCH GmbH

Supporting Information

Aggregation-Induced Emission Luminogens Sensitized Quasi-2D Hybrid Perovskites with Unique Photoluminescence and High Stability for Fabricating White Light-Emitting Diodes

Yuanwei Wang, Dingyuan Yan, Lei Wang, Dong Wang and Ben Zhong Tang**

Experimental section

Main Materials

Lead(II) bromide (99.999%), methylamine bromide (MABr, anhydrous, 99%), butylammonium (BA, 99%), HBr (99.95%), TFA (99.5%), DMSO (anhydrous, 99.8%), EtOH (anhydrous, 99.8%), MeCN (anhydrous, 99.8%), MeOH (anhydrous, 99.8%), DCM (anhydrous, 99.8%) and toluene (anhydrous, 99.8%) were obtained from Sigma-Aldrich. 4-picoline, *tert*-butyl (3-bromopropyl)carbamate, 4-Bromo-*N*,*N*-diphenylaniline, (5-formylthiophen-2-yl)boronic acid, piperidine, Pd(dppf)Cl_2, piperidine and K_2CO_3 were purchased from Energy, J&K or Meryer. All materials were used as received without further purification.

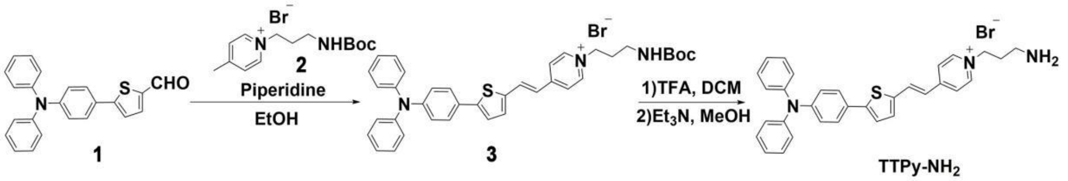

Scheme S1. Synthetic route of TTPy-NH_2.

Synthesis of 1: This intermediate was synthesized according to the literature method.[1] ^{1}H NMR (500 MHz, $CDCl_3$): 9.87 (s, 1H), 7.72 (d, *J* = 3.9 Hz, 1H), 7.54 (d, *J* = 8.8 Hz, 2H), 7.43 – 7.27 (m, 5H), 7.20–7.14 (m, 4H), 7.14–7.05 (m, 4H). ^{13}C NMR (126 MHz, $CDCl_3$): 182.55, 154.55, 149.14, 146.96, 141.32, 137.68, 129.47, 127.23, 126.13, 125.16, 123.86, 122.85, 122.35.

Synthesis of 2: A solution of 4-picoline (1.86 g, 20 mmol) and tert-butyl (3-bromopropyl) carbamate (4.8 g, 20 mmol) in acetonitrile (10 mL) was refluxed overnight and then cooled to room temperature. The reaction mixture was concentrated under reduced pressure and purified using DCM and methanol mixture (50:1 v/v) as eluting solvent to give the product as viscous oil (5.36 g, yield: 81%). ^{1}H NMR (500 MHz, DMSO-d_6): 8.99 (d, *J* = 6.3 Hz, 2H), 7.93 (d, *J* = 6.3 Hz, 2H), 6.85 (s, 1H), 4.53 (t, *J* = 7.1 Hz, 2H), 2.84 (d, *J* = 6.2 Hz, 2H), 1.93 (t, *J* = 6.9 Hz, 2H), 1.22 (s, 9H). ^{13}C NMR (151 MHz, DMSO-d_6): 159.24, 156.04, 144.33, 128.80, 78.22, 58.15, 37.03, 31.28, 28.67, 21.87. ESI HRMS: calcd. for $C_{14}H_{23}N_2O_2$ $[M-Br]^+$: 251.1754, found: 251.1754.

Synthesis of 3: A solution of **1** (71 mg, 0.2 mmol) and **2** (79.5 mg, 0.24 mmol) was refluxed under nitrogen in dry ethanol (10 mL) catalyzed by a few drops of piperidine overnight. After cooling to room temperature, the mixture was concentrated under

reduced pressure and purified by a neutral aluminum oxide column using DCM and methanol mixture (99:1 v/v) as eluting solvent to give the product as a red powder (110 mg, 82.4% of yield). 1H NMR (600 MHz, DMSO-d_6): 8.97 (d, J = 6.4 Hz, 2H), 8.29 (d, J = 16.0 Hz, 1H), 8.22 (d, J = 6.7 Hz, 2H), 7.67–7.61 (m, 2H), 7.52 (s, 2H), 7.41–7.33 (m, 4H), 7.17–7.11 (m, 3H), 7.10–7.07 (m, 4H), 7.02–6.96 (m, 2H), 4.53 (t, J = 7.1 Hz, 2H), 3.00 (q, J = 6.3 Hz, 2H), 2.06 (t, J = 6.8 Hz, 2H), 1.39 (s, 9H). 13C NMR (151 MHz, DMSO-d_6): 156.12, 153.12, 148.28, 147.75, 147.04, 144.62, 139.22, 134.62, 134.32, 130.22, 127.31, 126.80, 125.23, 124.69, 124.36, 123.72, 122.68, 121.66, 78.28, 57.91, 37.13, 31.25, 28.71. ESI HRMS: calcd. for $C_{37}H_{38}N_3O_2S$ $[M\text{-}Br]^+$: 588.2679, found: 588.2675.

Synthesis of TTPy-NH$_2$: To a solution of **3** (6.7 mg, 0.1 mmol) in DCM (1 mL) was added TFA (1 mL) 0 °C. The reaction mixture was slowly warmed to ambient temperature. The reaction was completed in 1 h by TLC analysis and the mixture was concentrated under reduced pressure. The obtained mixture was redissolved in methanol (1 mL) and triethylamine (100 uL), stirring for another 3 h. The final product was precipitate by adding diethyl ether to give a red powder (5.4 mg, 95% of yield). ^{1}H NMR (500 MHz, MeOD-d_4): 8.76–8.67 (m, 2H), 8.13–8.04 (m, 3H), 7.58–7.52 (m, 2H), 7.45 (d, J = 3.9 Hz, 1H), 7.35 (d, J = 3.9 Hz, 1H), 7.33–7.24 (m, 4H), 7.12–7.02 (m, 7H), 7.01–6.97 (m, 2H), 4.61 (t, J = 7.6 Hz, 2H), 3.09 (t, J = 7.7 Hz, 2H), 2.42–2.29 (m, 2H). ^{13}C NMR (126 MHz, MeOD-d_4): 154.38, 149.24, 148.62, 147.15, 143.49, 138.73, 135.10, 134.19, 129.22, 126.70, 126.53, 124.81, 123.57, 123.43, 123.33, 122.23, 120.09, 56.88, 36.03, 28.63. ESI HRMS: calcd. for $C_{32}H_{30}N_3S$ $[M\text{-}Br]^+$: 488.2155, found: 488.2154.

***Synthesis of TTPy-NH$_2$/PVK Nanosuspension*:** In a general procedure, 0.01 mmol $PbBr_2$, x mmol MABr, y mmol TTPy-NH$_2$ and 0.01 mmol HBr were dissolved in 200 µL DMSO. The mixture was rapidly injected into 5 mL toluene with vigorous stirring, and then nanosuspension of TTPy-NH$_2$/PVK was obtained. For n = 1 (2, 3 and 4), the x and y were respectively 0.0055 (0.0063,0.0069 and 0.0073), 0.0042 (0.0038, 0.0030 and 0.0026). BA/PVK nanosuspension was prepared in similar way by replacing TTPy-NH$_2$ with BA.

Preparation of TTPy-NH$_2$/PVK Films: The films were fabricated on the quartz substrates, which were cleaned sequentially with acetone, ethanol, and ultrapure water, ultrasonicated in each solvent for 10 min. To prepare TTPy-NH$_2$/PVK films, PVK precursor solutions of DMSO were spin-coated at 3000 rpm for 30 s and then annealed at 100 °C for 10 min to remove residual solvent and to crystallize the films.

***Fabrication of Pe-wLEDs:*[2]**

In the packaging procedure, 0.2 mL of BA/PVK in toluene was mixed with 200 mg resin. Then the resulting mixture was vacuumed for 0.5 h to remove the toluene and bubbles. The pure BA/PVK-resin was obtained. After that, the mixture was dropped onto a 365 nm chip and thermally cured for 2 h at 100 °C in an oven after the bubbles were removed. The prepared TTPy-NH$_2$/PVK film was attached on the top of package to achieve a on-chip PVK device.

Instruments

^{1}H NMR and ^{13}C NMR data were recorded on Bruker nuclear resonance (500 MHz and 600MHz) spectrometers unless otherwise specified, respectively. Chemical shifts (δ) are given in ppm relative to TMS. HRMS (ESI) analysis was performed by The Analytical Instrumentation Center at Peking University, Shenzhen Graduate School and (HRMS) data were reported with ion mass/charge (m/z) ratios as values in atomic mass units. Absorption spectra were measured on PerkinElmer Lambda 950 spectrophotometer. Photoluminescence (PL) spectra were recorded on Edinburgh FS5 spectrofluorometer. The absolute PL quantum yields (PLQYs) was determined by Hamamatsu quantum yield spectrometer C11347 Quantaurus QYwith an integrated sphere. The PL decay curves were collected with a time-resolved fluorescence spectrofluorometer (Deltaflex, Horiba). Atomic Force Microscope (AFM) was measured on Bruker Dimension ICON. AFM tapping mode (noncontact) was used to avoid sample damage, which can lead to a minor overestimation. Transmission electron microscope (TEM), HRTEM, SAED and mapping were measured on JEM F200 transmission electron microscope instruments. X-ray diffraction (XRD) pattern was measured on Bruker D8 Advance X-ray Diffractometer at 40 kV and 40 mA using Cu K_α radiation (λ = 1.5406 Å). The electroluminescent properties of the fabricated Pe-wLEDs were determined by using an integrating sphere spectroradiometer system (LHS-1000, Everfine) with an analyzer system (Everfine Photo-EINFO Co., Ltd.).

NMR and HRMS spectra of TTPy-NH_2 and intermediate products

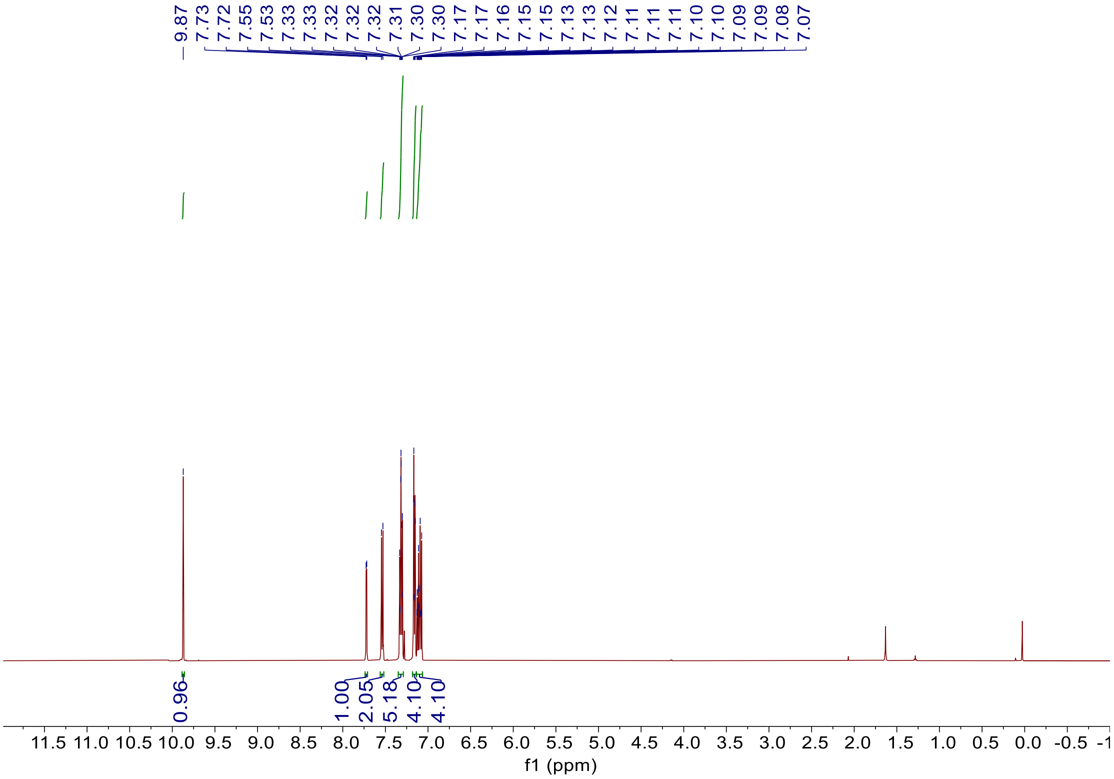

Figure S1. ^{1}H NMR spectrum of **1**.

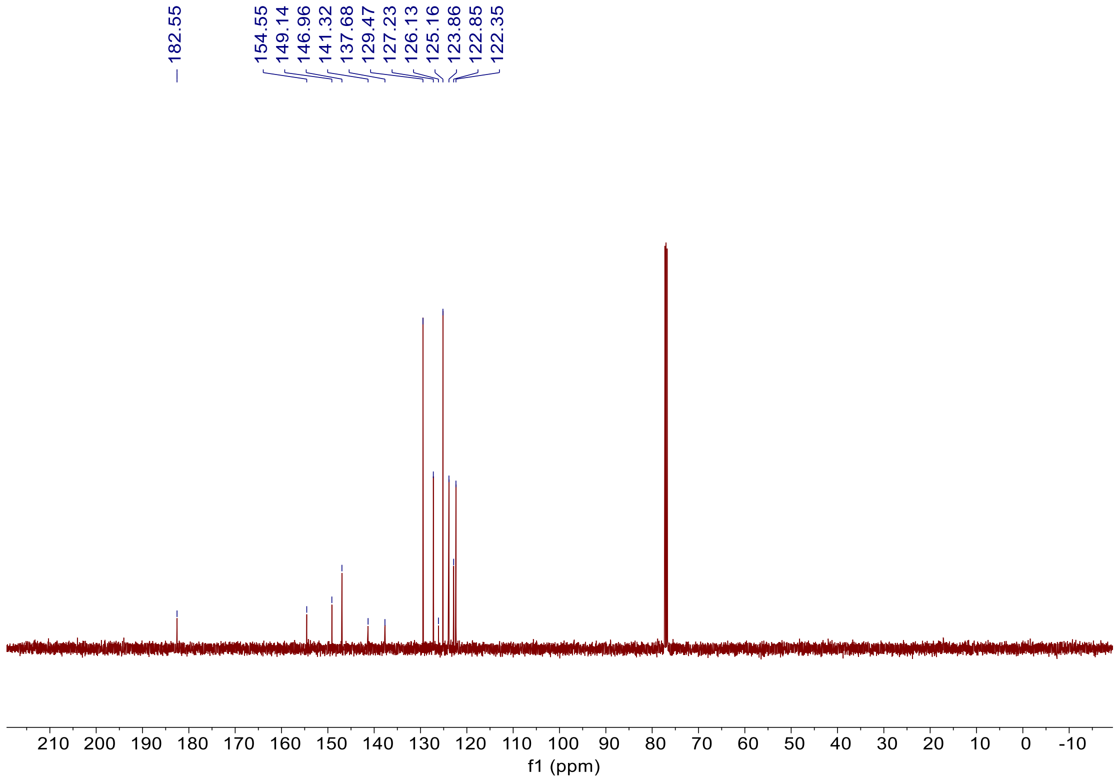

Figure S2. ^{13}C NMR spectrum of **1**.

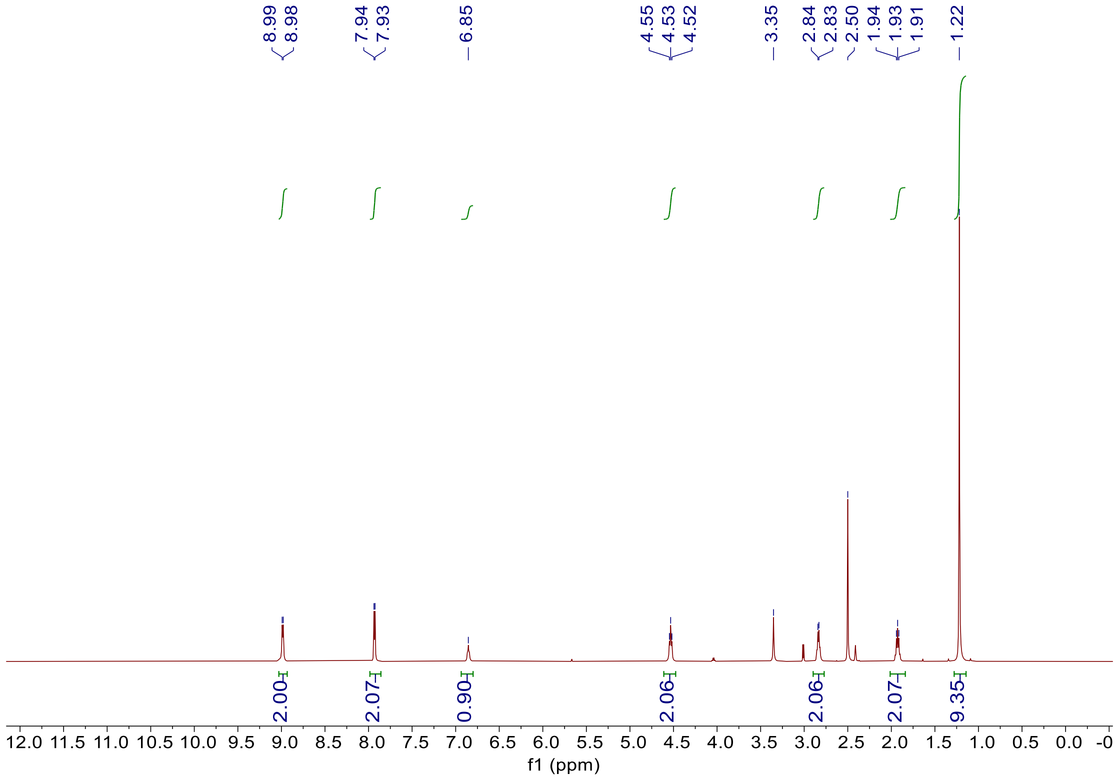

Figure S3. ^{1}H NMR spectrum of **2**.

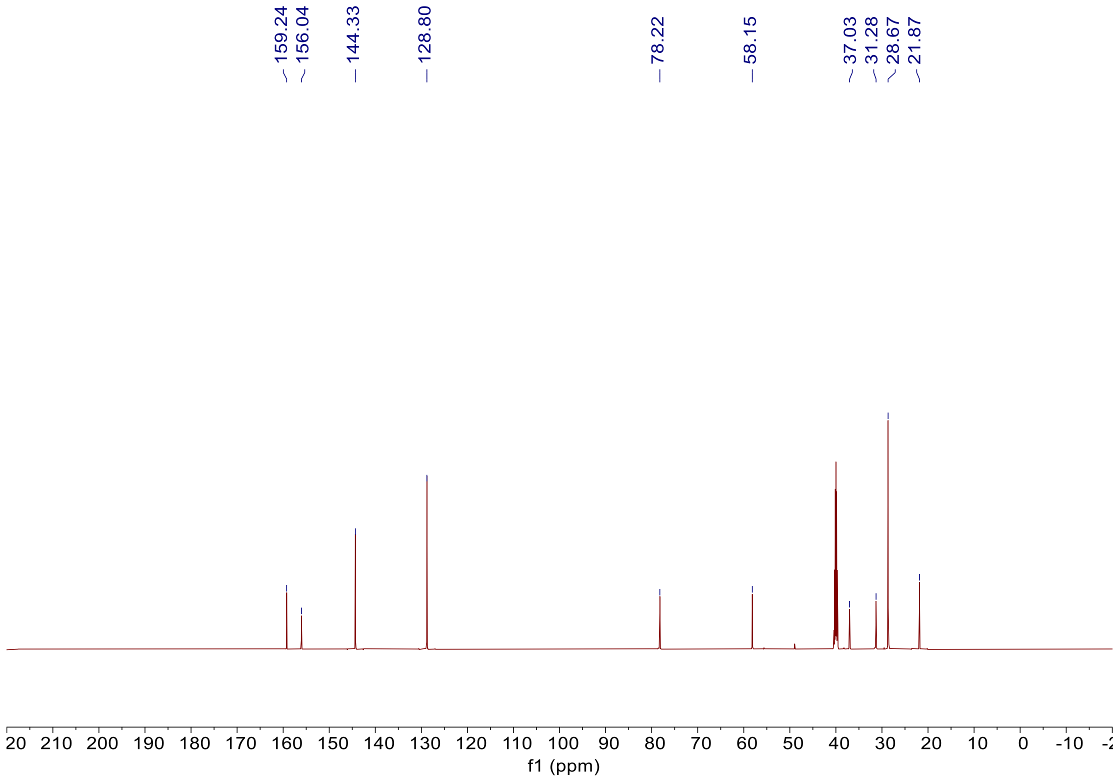

Figure S4. ^{13}C NMR spectrum of **2**.

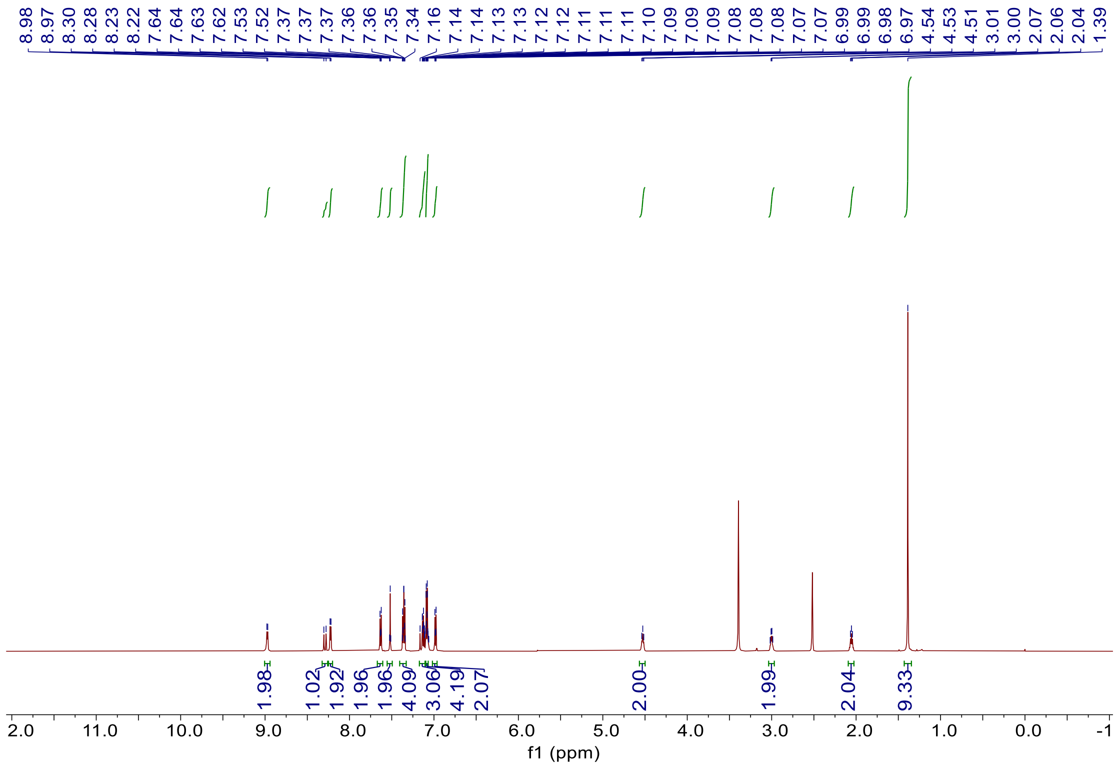

Figure S5. ^{1}H NMR spectrum of **3**.

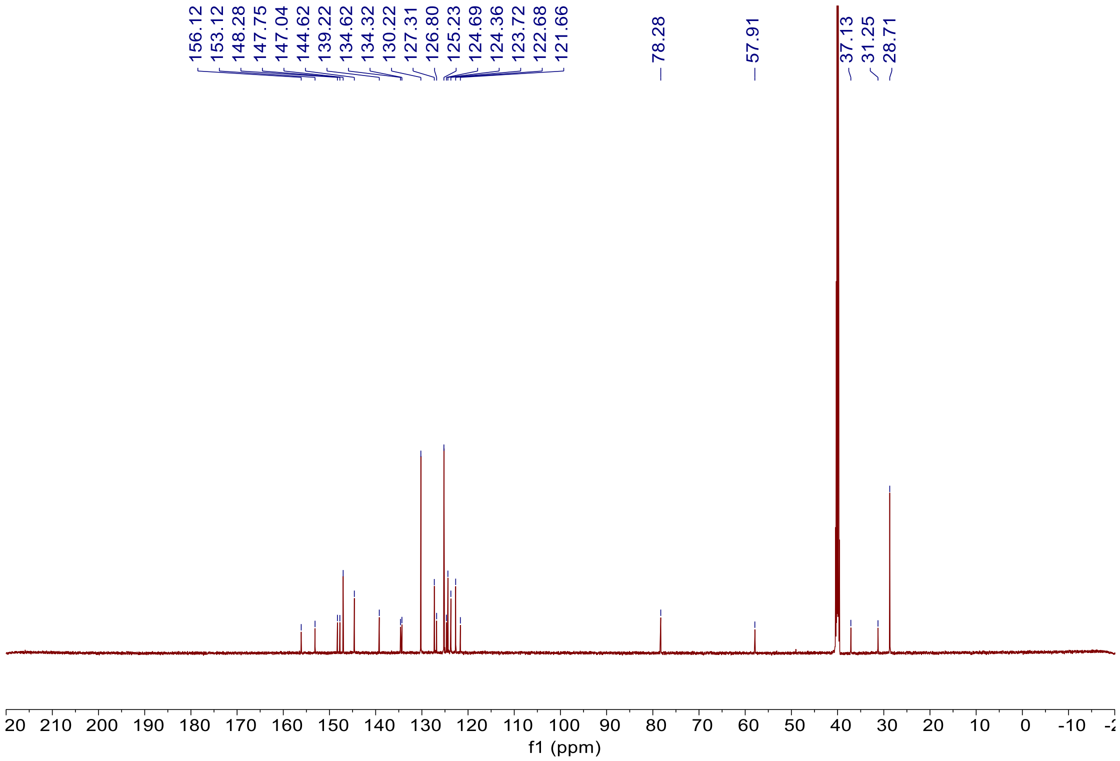

Figure S6. ^{13}C NMR spectrum of **3**.

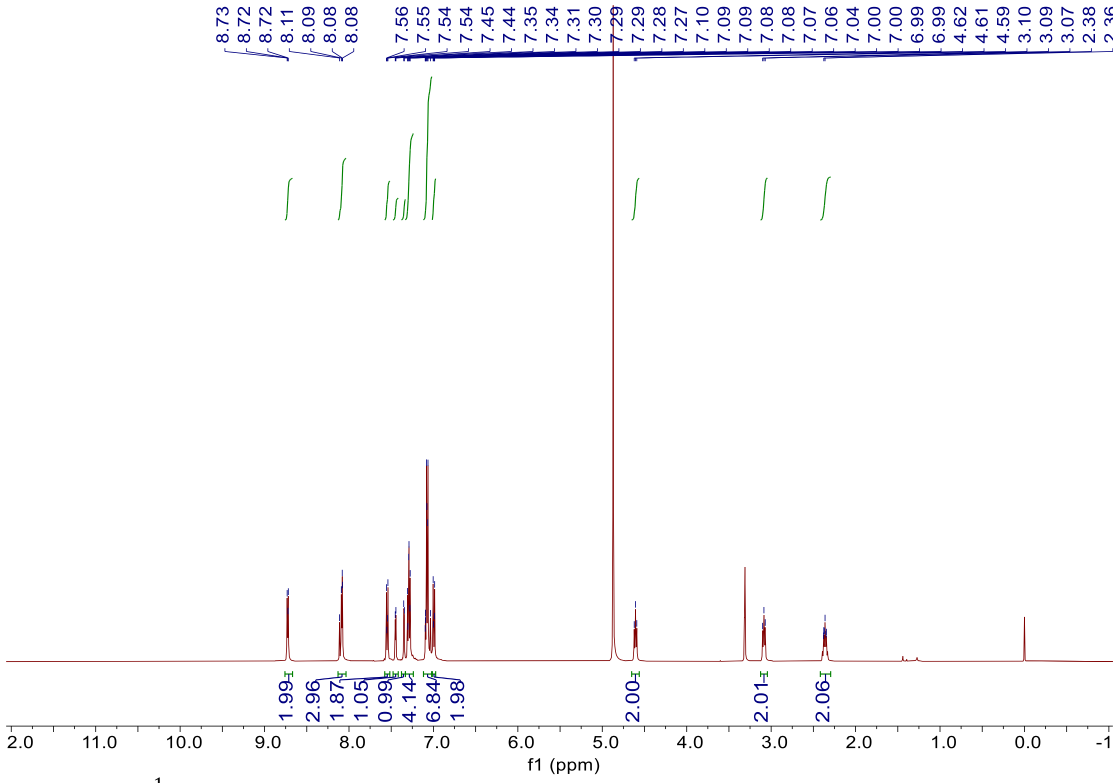

Figure S7. ^{1}H NMR spectrum of TTPy-NH_2.

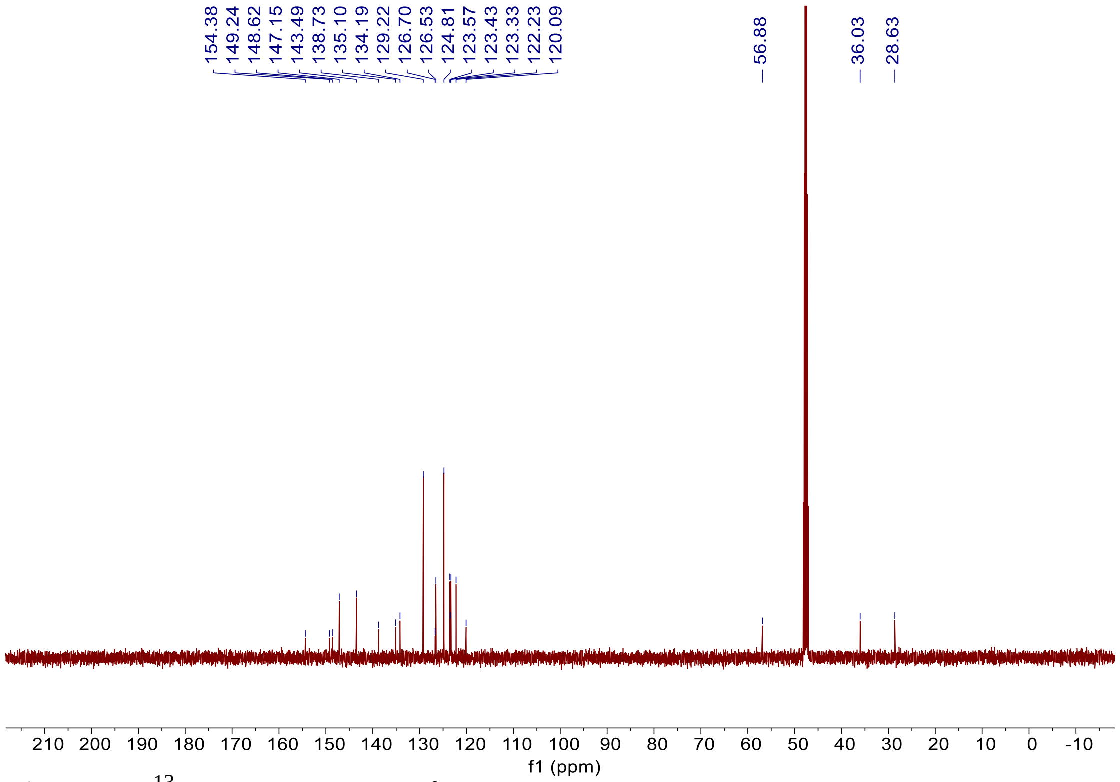

Figure S8. ^{13}C NMR spectrum of TTPy-NH_2.

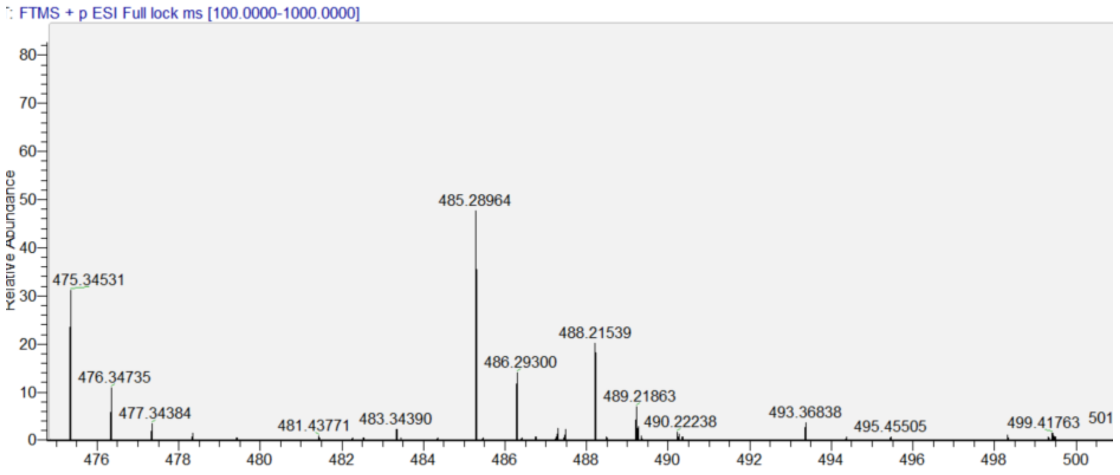

Figure S9. HRMS spectrum of TTPy-NH_2.

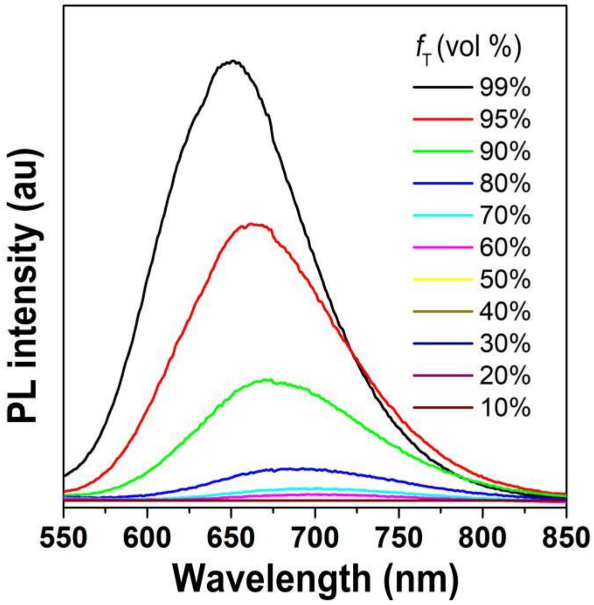

Figure S10. PL spectra of TTPy-NH_2 in DMSO/toluene with different toluene fractions f_T; Concentration: 10 μM; λ_{ex}: 482 nm.

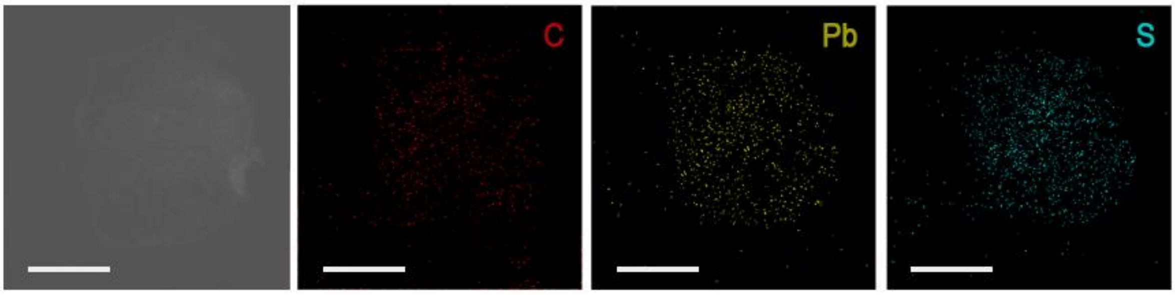

Figure S11. Electron diffraction pattern of TTPy-NH_2/PVK sheet, showing the elemental distribution of carbon, lead, and sulfur, respectively. Scale bars, 500 nm.

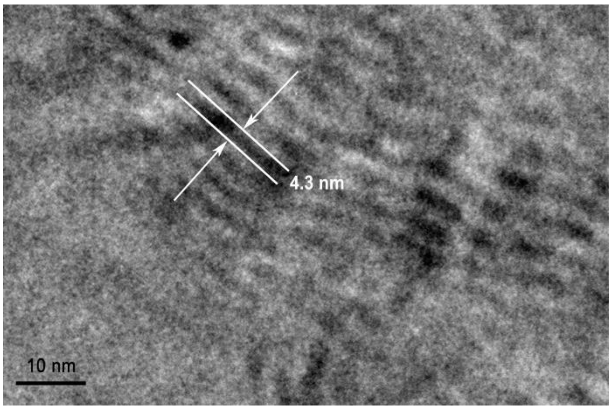

Figure S12. HRTEM image of stacks of TTPy-NH_2/PVK (n = 4) sheets.

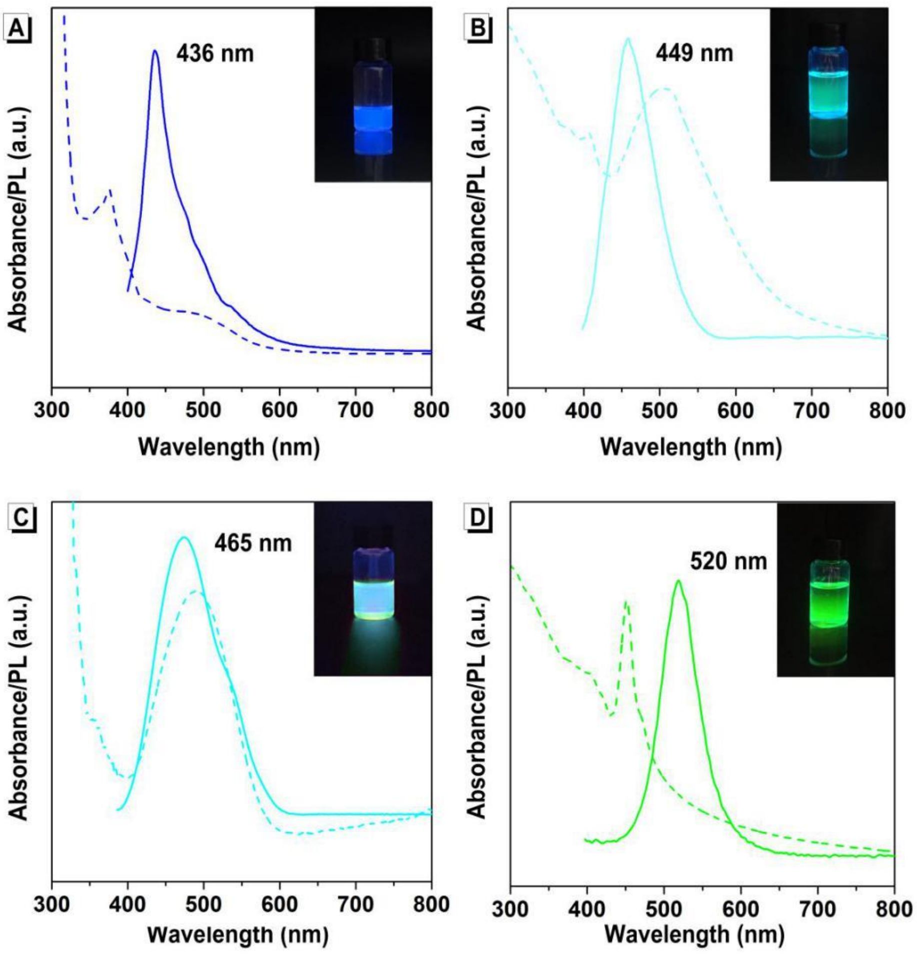

Figure S13. Absorption and emission spectra of A) TTPy-NH_2/PVK (n = 1), B) TTPy-NH_2/PVK (n = 2), C) TTPy-NH_2/PVK (n = 3) and D) bulk BA/PVK nanosuspension. Inset shows their photograph upon 365 nm excitation.

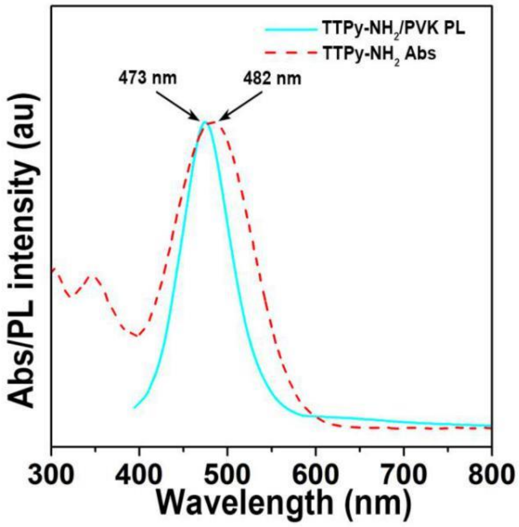

Figure S14. The spectral overlap of the PL spectrum of neat donor (blue solid) and absorption spectrum of neat acceptor (red dash).

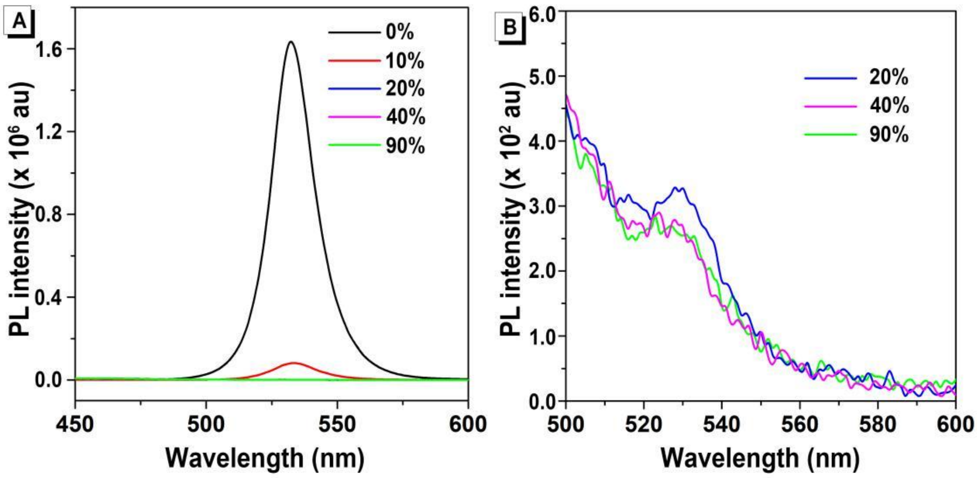

Figure S15. A) PL spectra of BA/PVK nanosuspension in DMSO/toluene with different DMSO fractions f_D; Concentration: 10 μM; λ_{ex}: 365 nm. B) Enlarged spectra of Figure A, where f_D was 20%, 40% and 90%.

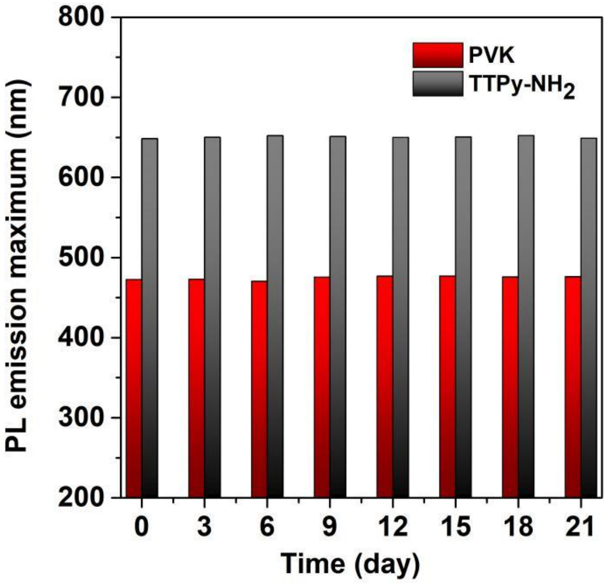

Figure S16. PL emission maximum stability of TTPy-NH_2/PVK film.

Table S1. Optical properties of AIEgens TTPy-NH_2.

	λ_{abs}[a] (nm)	λ_{em} (nm)			α_{AIE}
		Soln (Φ_F)[b,c]	Aggr (Φ_F)[c]	Solid (Φ_F)[c]	($I_{aggr,max}/I_{soln}$)
TTPy-NH_2	482	699 (0.2%)	651 (9.8%)	656 (10.6%)	60

[a] Absorption maximum in DMSO. [b] Emission maximum in DMSO (10 μM). [c] Fluorescence quantum yield determined by a calibrated integrating sphere.

Table S2. Optical properties of TTPy-NH_2/PVK under different excitation.

	Soln (Φ_F)[a]	Film (Φ_F)[b]
365 nm excitation	56.4%	62.2%
482 nm excitation	24.3%	29.5%

[a] Fluorescence quantum yield of TTPy-NH_2/PVK in toluene. [b] Fluorescence quantum yield of TTPy-NH_2/PVK film.

Table S3. Optical properties of TTPy-NH_2/PVK nanosuspension under different excitation.

	τ_1 (ns)[a]	τ_2 (ns)	τ_3 (ns)	τ_{ave} (ns)
Neat donor	23.73 (41.06%)	161.16 (46.16%)	4.99 (12.78%)	24.39
Donor in complex	11.25 (41.95%)	192.16 (42.6%)	3.46 (15.45%)	20.57
Neat acceptor	0.24 (36.8%)	0.98 (56.66%)	2.53 (6.55%)	0.46
Accepter in complex	0.83 (6.23%)	1.37 (60.62%)	3.18 (33.15%)	0.77

[a] Fluorescence lifetime and their relative amplitude, measured under ambient conditions. We measured the decay traces of donor emission in the complex by detecting emission at 473 nm with a bandpass of 1.5 nm, which allows emission bands at 473 nm (±5 nm) to pass, while cutting the rest of the emission that occurs below or above 473 nm. Similarly, the decay traces of accepter emission in the complex were measured by detecting emission at 656 nm.

Table S4. An overview of photoluminescence features of the white-emitting 2D perovskites.

Perovskite	FWHM (nm)	PLQE (%)	x/y CIE	CRI	CCT(K)	Ref.
(N-MEDA)$PbBr_4$	165	0.5	0.36/0.41	82	4669	3
(EDBE)$PbBr_4$	215	9	0.33/0.33	84	3990	4
(α-DMEN)$PbBr_4$	183	-	0.28/0.36	73	7843	5
$EA_4Pb_3Cl_{10}$	151	-	0.27/0.39	66	7720	6
DMPDA]$PbCl_4$	104	4.9	0.22/0.25	75	43787	7
(γ-MPA)$_2PbBr_4$	-	6.85	0.22/0.23	85	53281	8
(2meptH_2)$PbCl_4$	209	1.05	0.39/0.44	84	3958	9
TTPy-NH_2/PVK	-	4.8	0.329/0.353	89	6173	This work

Reference

[1] Y. Li, T. Ren, W. J. Dong, *J. Photochem. Photobiol. B* **2013**, *251*, 1.
[2] S. Yuan, Z.-K. Wang, L.-X. Xiao, C.-F. Zhang, S.-Y. Yang, B.-B. Chen, H.-T. Ge, Q.-S. Tian, Y. Jin, L.-S. Liao, *Adv. Mater.* **2019**, *31*, 1904319.
[3] E. R. Dohner, E. T. Hoke, H. I. Karunadasa, *J. Am. Chem. Soc.* **2014**, *136*, 1718.
[4] E. R. Dohner, A. Jaffe, L. R. Bradshaw, H. I. Karunadasa, *J. Am.Chem. Soc.* **2014**, *136*, 13154.
[5] L. Mao, Y. Wu, C. C. Stoumpos, M. R. Wasielewski, M. G. Kanatzidis, *J. Am. Chem. Soc.* **2017**, *139*, 5210.

[6] L. Mao, Y. Wu, C. C. Stoumpos, B. Traore, C. Katan, J. Even, M. R. Wasielewski, M. G. Kanatzidis, *J. Am. Chem. Soc.* **2017**, *139*, 11956.
[7] C.-Q. Jing, J. Wang, H.-F. Zhao, W.-X. Chu, Y. Yuan, Z. Wang, M.-F. Han, T. Xu, J.-Q. Zhao, X.-W. Lei, *Chem. Eur. J.* **2020**, *26*, 10307.
[8] Y. Li, Ch. Ji, L. Li, S. Wang, S. Han, Y. Peng, S. Zhang, J. Luo, *Inorg. Chem. Front.* DOI: 10.1039/D0QI01446J.
[9] S. Wang, Y. Yao, Z. Wu, Y. Peng, L. Li, J. Luo, *J. Mater. Chem. C* **2018**, *6*, 12267.
